# Flexible Synaptic Memristors With Controlled Rigidity in Zirconium‐Oxo Clusters for High‐Precision Neuromorphic Computing

**DOI:** 10.1002/advs.202412289

**Published:** 2025-01-24

**Authors:** Jae‐Hyeok Cho, Suk Yeop Chun, Ga Hye Kim, Panithan Sriboriboon, Sanghee Han, Seung Beom Shin, Jeehoon Kim, San Nam, Yunseok Kim, Yong‐Hoon Kim, Jung Ho Yoon, Myung‐Gil Kim

**Affiliations:** ^1^ School of Advanced Materials Science and Engineering Sungkyunkwan University Suwon 16419 Republic of Korea; ^2^ KU‐KIST Graduate School of Converging Science and Technology Korea University Seoul 02841 Republic of Korea

**Keywords:** flexible memristors, metal‐oxo clusters, neuromorphic computings, synaptic devices, Zr_6_O_4_OH_4_(OMc)_12_

## Abstract

Flexible memristors are promising candidates for multifunctional neuromorphic computing applications, overcoming the limitations of conventional computing devices. However, unpredictable switching behavior and poor mechanical stability in conventional memristors present significant challenges to achieving device reliability. Here, a reliable and flexible memristor using zirconium‐oxo cluster (Zr_6_O_4_OH_4_(OMc)_12_) as the resistive switching layer is demonstrated. The optimization of the structural rigidity of the hybrid oxo‐cluster network by thermal polymerization allows the precise formation of dispersed conductive cluster networks, enhancing the repeatability of the resistive switching with mechanical flexibility. The optimized memristor exhibits endurance of ∼10^4^ cycles and stable memory retention performance up to 10^4^ s, maintaining a high *I*_ON_/*I*_OFF_ ratio of 10^4^ under a bending radius of 2.5 mm. Moreover, the device achieves a pattern recognition accuracy of 97.44%, enabled by highly symmetric analog switching with multilevel conductance states. These results highlight that hybrid metal‐oxo clusters can provide novel material design principles for flexible and reliable neuromorphic applications, contributing to the development of artificial neural networks.

## Graphical abstract

The development of flexible synaptic memristors faces considerable challenges, including low cyclic endurance, limited on–off ratios, and asymmetry in conductance changes, while ensuring mechanical flexibility. This study demonstrates reliable synaptic characteristics with highly linear analog conductance modulation by utilizing a Zr_6_‐oxo cluster‐based memristor, highlighting their potential as advanced flexible memory components for artificial neural networks and neuromorphic computing applications.

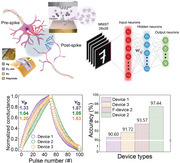

## 1 Introduction

With the recent advancement of next‐generation computing technologies, such as artificial intelligence and machine learning, a growing demand for more efficient processing of large amounts of data exists.^[^ ^1^, ^2^, ^3^ ^]^ However, the limitations of conventional computing concepts based on the von Neumann architecture are becoming increasingly apparent.^[^ ^4^, ^5^ ^]^ In this architecture, the physical separation between the central processing unit and the memory unit leads to significant energy and time costs in transferring data, referred to as the von Neumann bottleneck.^[^ ^6^, ^7^, ^8^ ^]^ To overcome these inherent limitations, emerging memory devices, such as resistive random‐access memory (ReRAM), ferroelectric random‐access memory, and phase‐change memory, have been explored to build neuromorphic computing architectures inspired by the synaptic structure of the human brain.^[^ ^9^, ^10^, ^11^ ^]^ Among these devices, ReRAMs, also known as memristors, are regarded as promising for neuromorphic applications that emulate the synaptic functions of biological neurons, owing to their high scalability, low power consumption, and high operating speed.^[^ ^12^, ^13^ ^]^

Memristors have a simple two‐terminal configuration, with the switching active layer sandwiched between an electrochemically active electrode (e.g., Ag, Cu) and an inert electrode (e.g., Au, Pt).^[^ ^14^, ^15^ ^]^ The mechanism for resistive switching between two distinct resistance states, i.e., a low‐resistance state (LRS) and high‐resistance state (HRS), is typically based on the formation and rupture of conductive filaments (CFs) between the two electrodes. Under a positive electric field, the atoms of the active metal are oxidized into ions. These ions migrate through the switching active layer, resulting in the formation of CFs, which corresponds to the LRS. The LRS can be switched to the HRS by applying an electric field of opposite polarity, which disrupts the CFs.

Unfortunately, achieving high‐performance and reliable operation of filamentary memristors has been challenging owing to the stochastic nature of filament formation and rupture.^[^ ^16^, ^17^, ^18^, ^19^ ^]^ The primary issue is the arbitrary growth of filaments in the switching active layer.^[^ ^20^ ^]^ Randomly created filaments can either remain permanently owing to excessive charge injection of metal ions or become thin and fragile in certain regions, leading to unpredictable switching behavior.^[^ ^16^ ^]^ Consequently, the dispersed conductive pathways created by these randomly formed filaments may interfere with reliable switching, particularly during repeated measurements.^[^ ^21^ ^]^ To address these challenges, numerous approaches have been reported to develop highly reliable memristor devices utilizing a wide range of materials, including oxides, 2D materials, nanomaterials, metal halides, and organic–inorganic hybrid materials.^[^ ^22^, ^23^, ^24^, ^25^, ^26^ ^]^ Despite extensive research efforts, the development of flexible memristors faces significant challenges in achieving reliable operation and high performance, such as low cyclic endurance, low on–off ratio, and asymmetry in conductance change, while maintaining mechanical flexibility. Consequently, new strategies aimed at optimizing the structural design of the material are essential for achieving ideal characteristics in flexible synaptic devices, including reliable and linear analog conductance modulation.

As a typical organic–inorganic hybrid material, metal‐oxo clusters (MOCs) are promising candidates for next‐generation electronic devices, exhibiting versatile functionalities owing to the unique characteristics resulting from the combination of inorganic and organic materials.^[^ ^27^ ^]^ Compared to other materials, MOCs offer a distinct advantage in terms of versatility in the fabrication process and straightforward synthesis, achieved through various combinations of inorganic MOC cores and organic ligands.^[^ ^28^ ^]^ Particularly, the ligands of MOCs can be precisely engineered to modulate their chemical, mechanical, and electrical properties, making them promising for next‐generation electronics such as high‐resolution nanopatterning and flexible electronic applications.

Herein, we report a highly reliable and flexible memristor device fabricated using zirconium‐oxo (Zr_6_‐oxo) clusters (Zr_6_O_4_OH_4_(OMc)_12_) as the switching active layer. The Zr_6_‐oxo clusters can be precisely controlled through a thermally activated polymerization process, which imparts structural rigidity and resists the formation of CFs or metal clusters. The switching mechanism of the Zr_6_‐oxo‐cluster‐based memristor is fundamentally driven by the formation of a 3D conductive cluster network rather than conventional filaments, enhancing the reliability and stability of the performance of the memory device. The optimized memristor exhibited highly reliable operation with an outstanding endurance of >10^4^ cycles, a high *I*_ON_/*I*_OFF_ ratio of 10^4^, and excellent retention characteristics up to 10^4^ s. Based on the nonvolatile memory properties of Ag/Zr_6_‐oxo/Au memristors, we successfully demonstrated a flexible memristor device on a flexible polyimide substrate with an endurance of >5 × 10^3^ cycles, an *I*_ON_/*I*_OFF_ ratio of 10^4^, and excellent retention characteristics up to 10^4^ s under a bending radius of 2.5 mm. Furthermore, reliable synaptic characteristics with highly linear analog conductance modulation were achieved, and the device exhibited a remarkable accuracy of 97.44% in pattern recognition simulation, revealing its potential as an advanced flexible memory component in artificial neural networks and neuromorphic computing.

## 2 Results and Discussions

**Figure 1:**
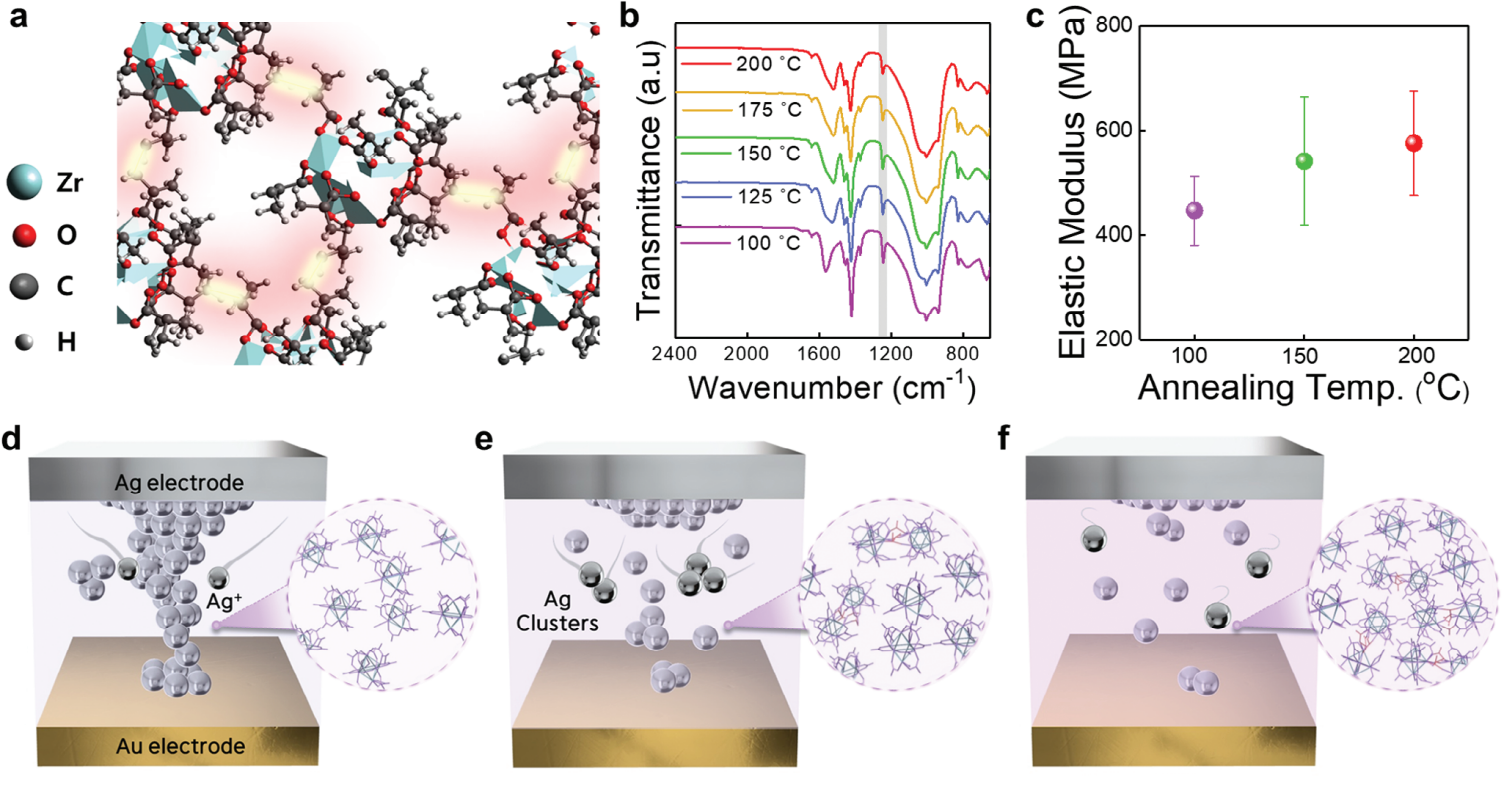
a) Schematic of the molecular structure and cross–linking reaction of Zr_6_O_4_OH_4_(OMc)_12_ clusters through the thermally activated polymerization process. b) FT‐IR results of the Zr_6_‐oxo cluster thin film under various thermal annealing conditions. c) Elastic modulus of the Zr_6_‐oxo cluster thin film at different annealing temperatures. Schematic of the diffusion and migration of silver cations in the Zr_6_‐oxo cluster thin film with d) low, e) intermediate, and f) high rigidity.

The molecular structure of a Zr_6_‐oxo cluster consists of an octahedral zirconium core coordinated by eight oxygen atoms, with methacrylate‐based ligands (OMc) (**Figure** **1**a). The Zr_6_‐oxo clusters can be cross–linked through thermal polymerization, facilitated by the presence of methyl methacrylate ligands containing C═ C bonds at the periphery of the clusters. The potential mechanism for the cross–linking reaction of the Zr_6_‐oxo cluster is depicted in Figure 1a. Mild heating activates the C═C bonds, generating reactive radical species that can easily induce cross–linking between the C═C bonds on the methacrylate ligands, leading to the formation of a cross–linked cluster network. Figure 1b shows the Fourier‐transform infrared spectroscopy (FT‐IR) results of the Zr_6_‐oxo cluster thin film under various thermal annealing conditions. The ligand shows three main infrared (IR) signals in the range of 650–1650 cm^−1^. Notably, the characteristic IR peak at 1246 cm^−1^, corresponding to the C═C bonding (C(CH_3_)═CH_2_) in the methacrylate ligands of the Zr_6_‐oxo cluster, decreased with increasing thermal annealing temperature due to the thermal cross–linking process. In addition, a noticeable shift in the diffraction peak position to a lower angle was observed in the grazing incidence X‐ray diffraction (GIXRD) patterns, indicating a transition from C═C bonds to C─C bonds resulting from the thermal cross–linking reaction (Figure S1, Supporting Information). These findings confirm that the Zr_6_‐oxo cluster undergoes cross–linking through thermal polymerization, consistent with a previous report.^[^ ^29^ ^]^

The material rigidity of the Zr_6_‐oxo cluster, utilized as a switching matrix, plays a crucial role in regulating ion diffusion and migration, thereby affecting the formation of internal conduction paths. A less rigid Zr_6_‐oxo cluster is expected to facilitate faster ionic diffusion, whereas the cross–linked Zr_6_‐oxo cluster, with increased structural rigidity, resists the formation of external metal filaments or clusters due to slower ionic diffusion and mechanical suppression of large Ag crystal growth within the matrix. Figure 1c shows the elastic modulus of the Zr_6_‐oxo thin film annealed at various temperatures. The higher modulus values suggest that Ag ion migration in the cluster matrix can be effectively controlled. The increase in the elastic modulus, as observed from the force–distance curves obtained via atomic force microscopy (AFM) (Figure S2, Supporting Information), with increasing temperature, further confirms a substantial enhancement in the mechanical rigidity of the Zr_6_‐oxo thin films. This increased rigidity significantly affects the formation of conduction paths in the Zr_6_‐oxo cluster switching matrix, as the enhanced mechanical constraint may inhibit the formation of continuous filaments, instead promoting dispersed and isolated Ag clusters across the film, as illustrated in Figure 1d–f.

**Figure 2:**
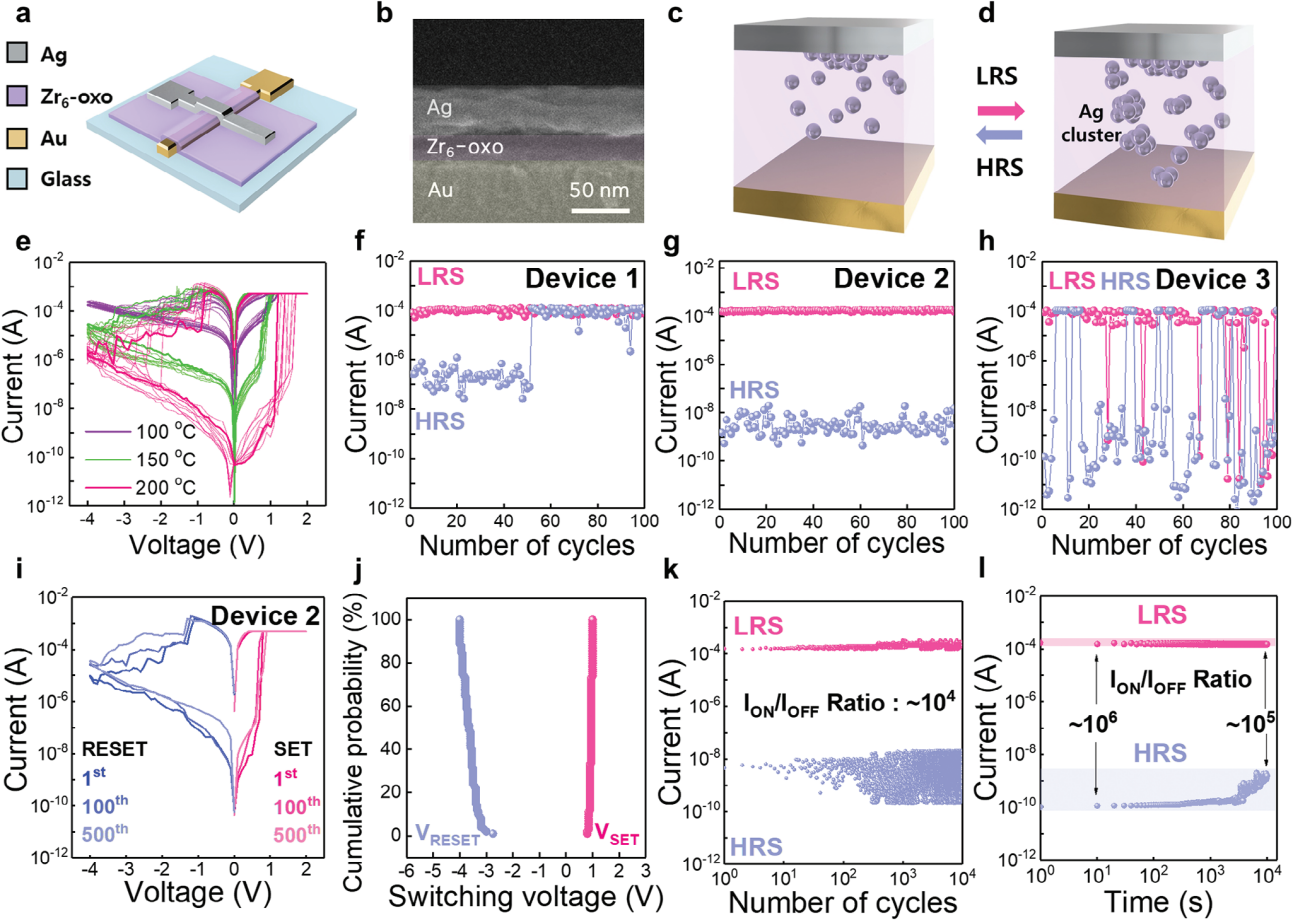
a) Schematic of the device structure featuring the Ag/Zr_6_‐oxo/Au vertical crossbar electrode configuration. b) Cross‐sectional SEM image of the Ag/Zr_6_‐oxo/Au device. Schematic representation of the switching mechanism in Zr_6_‐oxo‐cluster‐based memristors between the c) HRS and d) LRS. e) *I–V* characteristics of memristor devices under three different annealing conditions. Representative cyclic endurance tests for a f) device annealed at 100 °C (device 1), g) device annealed at 150 °C (device 2), and h) device annealed at 200 °C (device 3). i) *I–V* characteristics of device 2 over 500 DC sweep cycles. j) Cumulative probability distributions of SET and RESET voltages for cycle‐to‐cycle measurements. k) Resistance distribution in the HRS and LRS of device 2 over 10^4^ cycles. l) Memory retention characteristics of device 2.

To explore the potential application of the Zr_6_‐oxo cluster as a switching active layer, we designed a device structure with an Ag/Zr_6_‐oxo/Au vertical crossbar electrode configuration, illustrated in **Figure** **2**a. The cross‐sectional scanning electron microscopy (SEM) image in Figure 2b confirms the successful integration of each layer within the memristor device. The resistive switching mechanism of the Ag/Zr_6_‐oxo/Au device follows the typical electrochemical metallization process, depicted in Figure 2c,d. During a positive voltage sweep, Ag atoms oxidize into Ag^+^ ions, which migrate through the Zr_6_‐oxo matrix, slightly reducing its resistivity. Upon the subsequent reduction of Ag, conduction paths form within the Zr_6_‐oxo matrix, leading to an abrupt resistance switch from the HRS to LRS. These conduction paths, created by Ag clusters, are maintained after the SET operation even without external bias. When a negative voltage is applied, the device switches from the LRS to HRS due to the reversible redox reaction and dissolution of Ag filaments.^[^ ^31^ ^]^ Figure 2e shows the current–voltage (*I–V*) characteristics under various annealing conditions, exhibiting typical nonvolatile resistive switching behaviors during a DC voltage sweep. A compliance current of 5 × 10^−4^ A was set during the positive voltage sweep to prevent permanent breakdown of the devices. During the negative voltage sweep, the compliance current was released to dissolve the conductive paths, which required a higher current to break the Ag conductive pathways. Notably, resistive switching characteristics were observed under all the annealing conditions, indicating a correlation with the film's density (Figure S3, Supporting Information). As the annealing temperature increased, a noticeable decrease in the HRS (off‐current state) was observed, primarily due to the film densification, which restricted ion mobility and suppressed Ag crystal growth.^[^ ^32^ ^]^

To further confirm the repeatability of the resistive switching characteristics, we performed endurance measurements by applying a series of SET pulses (2 V with a duration of 5 ms) and RESET pulses (−4 V with a duration of 5 ms) to the device over 100 cycles. Specifically, devices annealed at 100 °C (referred to as device 1) exhibited significant vulnerability, undergoing permanent breakdown after only 50 cycles of voltage sweep, as shown in Figure 2f. This phenomenon suggests that lower annealing temperatures might compromise the ability of the device to maintain stable conduction pathways owing to the overgrowth of CFs. In contrast, device 3 (Figure 2h), which was annealed at 200 °C, exhibited random and unpredictable resistive switching characteristics. These fluctuations in the resistance state led to difficulties in defining the HRS and LRS, which are crucial for the reliable operation of memory devices. The challenge in establishing these distinct states arises from the insufficient formation of CFs, attributed to the restricted Ag ion diffusion and metallic Ag crystal growth at these higher annealing temperatures with increased mechanical rigidity. The device annealed at the optimized condition of 150 °C (device 2) demonstrated superior performance, showing reliable and stable switching behavior without any permanent breakdown (Figure 2g). This robust performance represents the optimal condition for ensuring the durability and reliability of the device by effectively modulating ion transportation, which directly contributes to the growth dynamics of the metal clusters.

Overall, the different switching characteristics among devices 1–3 were mainly due to variations in Ag diffusivity, resulting from differences in the ionic conductivity of Ag cations within the Zr_6_‐oxo thin film layers of different densities. Consequently, the reliability of the resistive switching characteristics is highly dependent on the kinetics of the ion transport process in the switching active layer. The results from the *I–V* characteristics of the three different devices in continuous DC sweep mode are consistent with the cyclic endurance data (Figure S4, Supporting Information). Figure 2i shows the *I–V* characteristics of the optimized device 2 over 500 repeated resistive switching cycles. The statistical data for the SET and RESET voltages are plotted in Figure 2j, showing a uniform SET/RESET switching voltage dispersion over 100 cycles. The SET voltage (*V*_SET_) is defined as the point where the current abruptly increases, indicating the transition to the low‐resistance state (LRS). Conversely, RESET voltage (*V*_RESET_) is defined as the point in the negative bias region where the current gradually decreases and reaches the fully “off” current state, corresponding to the high‐resistance state (HRS). The V_SET_ was observed to range from 0.8 to 1 V, whereas the *V*_RESET_ varied from −2.75 to −4 V. These switching voltages are strongly dependent on the thickness of the zirconium‐oxo cluster layer, as they directly influence the electric field required for ion migration. By optimizing the thickness of the zirconium‐oxo cluster layer, the memory window and operating voltage can be precisely adjusted (Figure S5, Supporting Information). The endurance test, conducted using a series of SET pulses (2 V with a duration of 5 ms) and RESET pulses (−4 V with a duration of 5 ms) with a read voltage of 0.1 V, showed stable resistive switching operation with a high *I*_ON/OFF_ ratio of 10^4^ over >10^4^ cycles (Figure 2k). Additionally, both the HRS and LRS were maintained without memory degradation for up to 10^4^ s with a high I_ON/OFF_ ratio of 10^5^ (Figure 2l). To further evaluate the impact of temperature variations on device 2, measurements were conducted after raising the temperature to 50 °C to simulate thermal effects caused by consecutive operational cycles. As shown in Figure S6 (Supporting Information), the device maintained consistent resistive switching behavior with no significant changes in *V*_SET_, *V*_RESET,_ and *I*_ON/OFF_ ratio. These results suggest that adjusting the cross–linking density of the resistive switching matrix based on the Zr_6_‐oxo cluster layer through thermal cross–linking is an effective strategy for achieving highly reliable resistive switching with enhanced cyclic endurance.

**Figure 3:**
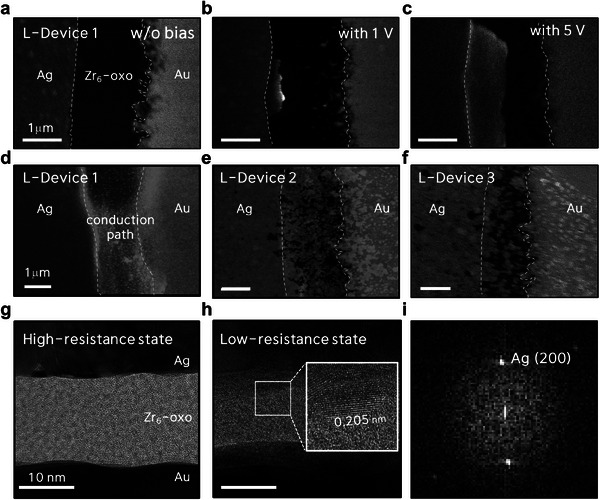
FE‐SEM images of lateral device 1 (L‐device 1) designed with a near‐microscale gap between the two electrodes, subjected to a voltage bias of a) 0 V, b) 1 V, and c) 5 V. FE‐SEM images showing the LRS of devices under various annealing conditions: d) L‐device 1 annealed at 100 °C, e) L‐device 2 annealed at 150 °C, and f) L‐device 3 annealed at 200 °C. Cross‐sectional high‐resolution TEM image of vertical memristor device 2 in the g) HRS and h) LRS. The inset shows Ag clusters embedded in the Zr_6_‐oxo cluster layer. i) FFT pattern of the specific area shown in the inset of (h).

To directly investigate the growth structure of the Ag conductive path in the Zr_6_‐oxo cluster matrix, we fabricated lateral devices (L‐device n, *n =* 1–3 corresponds to 100, 150, and 200 °C, respectively) specially designed with a near‐microscale gap between the two electrodes (**Figure** **3**a). The formation dynamics of the Ag conductive paths were observed using field‐emission SEM (FE‐SEM). As the positive voltage bias increased, the conduction path between the Ag and Au electrodes, originating from Ag nucleation, gradually became larger and thicker (Figure 3b,c). Consequently, the subsequent growth of the Ag nucleation is physically connected to the inert electrode, leading to LRS conductance. More importantly, the growth of the conduction paths varied significantly among the devices subjected to different annealing temperatures. For L‐device 1, the conduction path constituted a relatively large portion of the overall filament growth (Figure 3d). In contrast, in L‐device 2, the conduction path appeared narrower (Figure 3e), due to its densely cross–linked network with enhanced rigidity, which resulted from the higher annealing temperature of the Zr_6_‐oxo matrix. On the other hand, for L‐device 3, filament growth appeared more difficult, likely hindered by suppressed cation mobility, resulting in incomplete conduction paths (Figure 3f). The Ag nucleation in the Zr_6_‐oxo cluster layer, structurally tuned by thermally activated polymerization, might have promoted the formation of Ag metal clusters rather than excessively grown conductive paths. These Ag metal clusters within the cross–linked Zr_6_‐oxo matrix are expected to exist in discrete, localized regions, suggesting the potential for multiple resistance states. To confirm the feasibility of multilevel programming in our device, the compliance current was incrementally adjusted in a positive voltage step, achieving multiple LRS conductance levels (Figure S7, Supporting Information). Additionally, multiple HRS conductance levels were observed through controlled RESET stop voltage (*V*_stop_). As *V*_stop_ increased, the corresponding current level decreased in the retention curve, confirming the capability of achieving multiple HRS levels through controlled reset voltages. This behavior is further supported by FE‐SEM images (Figure S8, Supporting Information), which illustrate the spatial distribution of Ag clusters under different reset voltage bias conditions. The formation of Ag metal clusters, facilitated by the controlled structural rigidity of the Zr_6_‐oxo cluster, is believed to enhance the repeatability and stability of the resistive switching behavior.

To verify Ag nucleation in the Zr_6_‐oxo thin film layer, bright‐field transmission electron microscopy (TEM) imaging was performed on the optimized memristor device 2 in both the HRS and LRS. For the Zr_6_‐oxo layer in the HRS, Figure 3g shows the vertical structure of the Ag/Zr_6_‐oxo/Au device, without noticeable Ag nucleation inside the Zr_6_‐oxo layer. However, in the lattice‐resolved TEM image and its fast Fourier transform (FFT) pattern in Figure 3h,i, obtained after applying a voltage bias of 2 V to the Ag electrode, lattice fringes with a spacing of 0.205 nm were observed in the Zr_6_‐oxo layer, indicating Ag nucleation growth along the Ag (200) crystal plane. Furthermore, we observed a shortening of the effective distance between the two electrodes, resulting from the growth of Ag ions at the surface of the counter electrode.^[^ ^32^, ^33^, ^34^ ^]^

**Figure 4:**
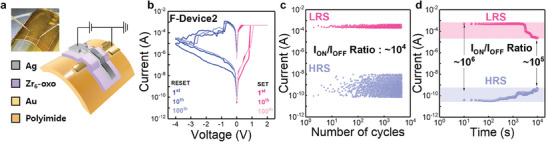
a) Schematic of the flexible memristor with the Ag/Zr_6_‐oxo/Au device on a polyimide substrate. b) *I–V* characteristics of F‐device 2 over 100 DC sweep cycles. c) Resistance distribution in the HRS and LRS of the flexible device over 5000 cycles. d) Memory retention characteristics of the flexible device under bending‐induced strain of 1.0% (bending radius of 2.5 mm).

Based on a comprehensive understanding of the resistive switching properties of the Zr_6_‐ oxo clusters, a flexible Ag/Zr_6_‐oxo/Au device was fabricated on a polyimide substrate, as shown in **Figure** **4**a. The electrical performance of the flexible device, based on device 2, which demonstrated highly reliable cyclic endurance, was evaluated in a bent state. Figure 4b shows the *I–V* characteristics of the device over 100 consecutive DC sweep cycles. The flexible device consistently exhibited nonvolatile resistive switching behaviors, consistent with the results obtained from the flat device. In addition, the flexible device showed robust resistive switching performance under bending‐induced strains of 1.0%, 0.5%, and 0.25%, corresponding to bending radii of 2.5, 5, and 10 mm, respectively (Figure S9, Supporting Information). The stain stress was calculated using the following equation:

(1) $$\varepsilon =\frac{t_{s}}{2r}\;$$

where *t*_s_ and *r* represent the thickness of the substrate (50 µm) and the radius of curvature of the device in its bent state, respectively.^[^ ^49^ ^]^ Furthermore, the device maintained a high on–off ratio of up to 10^5^ after 500 bending cycles at a strain of 1.0% (bending radius of 2.5 mm), demonstrating excellent flexibility (Figure S10, Supporting Information). The device exhibited stable switching endurance, with no noticeable performance degradation, maintaining a high *I*_ON/OFF_ ratio of 10^4^ over 5 × 10^3^ cycles under a bending radius of 2.5 mm (Figure 4c). Retention tests further confirmed that both the HRS and LRS were sustained for up to 10^4^ s, with only slight performance degradation, while still achieving a high *I*_ON/OFF_ ratio of 10^5^ (Figure. 4d). Compared to other flexible memory devices reported in the literature, the high *I*_ON/OFF_ ratio of 10^4^ under a bending radius of 2.5 mm, along with stable endurance over 500 bending cycles, demonstrates exceptional reliability for flexible applications (Table S1, Supporting Information). These results confirm that the device operates reliably under various bending conditions with robust switching endurance, highlighting its potential for flexible memory applications.

**Figure 5:**
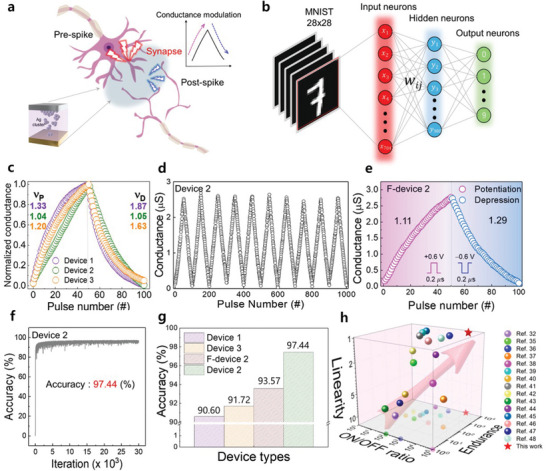
a) Schematic of a biological synapse illustrating the concept of synaptic modulation using a memristor‐based artificial synapse. b) Schematic of an MNIST‐data‐based neural network simulation system. c) Analog conductance modulations for three different device conditions under 0.8/−0.8 V, 200 ns pulse conditions. d) Ten repeatable sequential conductance modulations for device 2 performed under 0.8/−0.8 V, 200 ns pulse conditions. e) Analog conductance modulations for F‐device 2 under 0.6/−0.6 V, 200 ns pulse conditions. f) Accuracy of pattern recognition for device 2 after 30 000 iterations. g) Comparison of pattern recognition accuracy under different conditions after 30 000 iterations. h) Comparison of various memristor devices in terms of linearity, *I*_ON/OFF_ ratio, and endurance. The red star represents the performance of the proposed device in this study, demonstrating superior linearity and endurance compared to the referenced devices.

Biological synapses play a crucial role in the learning process by responding to various external stimuli within the nervous system. Synaptic weights represent the strength of the connection between two consecutive synaptic neurons and are updated in an analog manner based on learning rules. This occurs as stimuli from the pre‐neuron reach the synaptic cleft and are transmitted to the post‐neuron (**Figure** **5**a). Figure 5b illustrates a schematic of a neural network for MNIST pattern recognition, consisting of three neuron layers with 784 input neurons, 300 hidden neurons, and 10 output neurons. In neuromorphic computation, achieving high linearity and symmetry in the potentiation and depression of conductance modulation is essential for ensuring the desired inference accuracy.

To investigate the synaptic characteristics of devices 1, 2, and 3, which are based on metal clustering ion‐migration dynamics, conductance modulation was performed using a train of 50 consecutive positive pulses with an amplitude of 0.8 V, followed by 50 consecutive negative pulses with an amplitude of −0.8 V, both with pulse widths of 200 ns (Figure 5c). To evaluate linearity, we defined the linearity factor as follows:

(2) $$\mathrm{Linearity}\;\mathrm{factor}\;=\left(\frac{\Delta G_{max\;}+\Delta G_{min}}{2}\right)/\left(\frac{G_{max}\;-\;G_{min}}{pulse\;\;number}\right)$$

where Δ*G*_max _and Δ*G*_min _are the maximum and minimum changes in conductance between adjacent levels, respectively. The calculated linearity values for potentiation and depression were 1.33 and 1.87, respectively, for device 1; 1.04 and 1.05, respectively, for device 2; and 1.20 and 1.63, respectively, for device 3. Device 2 exhibited the most linear and symmetric conductance updates under identical pulse conditions, confirming its exceptional linearity. In contrast, devices 1 and 3 showed deviations from the ideal value of 1 during both potentiation and depression, indicating nonlinear and asymmetrical conductance updates. Figure 5d shows the stable repetitive behavior of 10 sequential conductance modulations, demonstrating reliable and predictable conductance updates in response to a series of potentiation and depression pulses. Additionally, under the conditions of device 2 on a flexible substrate (F‐device 2), the calculated linearity values for potentiation and depression were 1.11 and 1.29, respectively, demonstrating high linearity comparable to those observed on a rigid substrate (Figure 5e).

To further evaluate the performance of the Ag/Zr_6_‐oxo/Au device for neuromorphic computing applications, we conducted a neural network simulation for MNIST pattern recognition, as shown in Figure 5b. The conductance modulation performances were modeled using the following equation:

(3) $$G\left(n\right)=A+Be^{-Cn}$$

where the pulse index, *n*, is evaluated before the synaptic weight update for each epoch. The ideal parameters A, B, and C were extracted by curve‐fitting the experimentally obtained conductance modulation data shown in Figure 5c. The MNIST dataset, comprising images of handwritten digits with 28 × 28 grayscale pixels, included 60 000 training samples and 10 000 validation samples. Synaptic weights applied between each neuron layer were converted from the conductance values in Figure 5c and expressed as *w =* (*G* − *G*_mean_)/ *G*_diff_, where *G*_mean =_ [*G*_P_ (0) + *G*_D_ (0)] / 2 and *G*_diff_ = *G*_D_ (0) + *G*_P_ (0). A training session was conducted for 30 000 iterations. The maximum inference accuracies of devices 1 and 3 were 90.60% and 91.72%, respectively (Figure S11, Supporting Information). In contrast, device 2 exhibited a superior accuracy of 97.44% (Figure 5f). Moreover, when device 2 was tested on a flexible substrate, it achieved a remarkable maximum inference accuracy of 93.57%. The maximum inference accuracy of device 2, on both standard and flexible substrates, was clearly higher than that of devices 1 and 3 (Figure 5g), indicating its excellent performance for high‐precision neuromorphic computing. The backpropagation algorithm assessed the weights by assuming a linear change in conductance, determining the number of programming pulses required to adjust the weights. This approach leverages high linearity and symmetry in the conductance modulation process to ensure high precision and minimal fluctuation. Compared to previously reported conductive bridge random‐access memory devices,^[^ ^32^, ^35^, ^36^, ^37^, ^38^, ^39^, ^40^, ^41^, ^42^, ^43^, ^44^, ^45^, ^46^, ^47^, ^48^ ^]^ the Ag/Zr_6_‐oxo/Au device shows significantly improved properties (Figure 5h). These advancements highlight the effectiveness of the proposed metal clustering ion‐migration dynamics. Key parameters such as endurance, on/off ratio, and linearity—critical for evaluating the reliability of synaptic devices—have been extensively studied, underscoring the superior performance of our device in various aspects (Table S2, Supporting Information).

## 3 Conclusions

In conclusion, we systematically investigated the potential of Zr_6_‐oxo clusters for the development of flexible memristor devices. The structural rigidity of the Zr_6_‐oxo cluster, optimized through a thermally activated polymerization process at 150 °C, allows for precise control over the growth dynamics of metal clusters. This control facilitates the formation of Ag metal clusters, enhancing resistive switching reliability by preventing excessive CF paths. The optimized device demonstrated exceptional repeatability in resistive switching with an endurance exceeding 10^4^ cycles and stable memory retention performance up to 10^4^ s, even under mechanical stress, while maintaining a high *I*_ON/OFF_ ratio. Furthermore, the device exhibited high reliability under mechanical bending with radii of 2.5, 5, and 10 mm. With highly linear analog conductance modulation, the memristor achieved a remarkable accuracy of 97.44% in pattern recognition simulations, revealing its potential as an advanced flexible memory component for artificial neural networks and neuromorphic computing. These results validate the robustness of the zirconium‐oxo‐cluster‐based memristor and highlight their potential in flexible memory applications for advanced computing systems.

## 4 Experimental Section

### Materials

According to previous reports (29–30), to synthesize Zr_6_O_4_OH_4_(OMc)_12_, 0.5 mL of ethanol (Sigma Aldrich, USA) was added to 4 mL of zirconium n‐propoxide (70 wt.% in n‐propanol) (Alfa Aesar, USA). Following this, 4 mL of methacrylic acid (TCI Chemicals, Japan) was rapidly injected into the zirconium solution. The resulting mixture was then stored for 14 d to obtain needle‐shaped Zr_6_O_4_OH_4_(OMc)_12_ crystals. After crystallization, the solution was decanted, and the crystals were dried using vacuum evaporation to remove any residual solvent. All procedures were carried out under an inert atmosphere.

### Device Fabrication

For the fabrication of vertical memristors with the Ag/Zr_6_‐oxo/Au crossbar electrode configuration, glass substrates were cleaned in acetone and isopropyl alcohol using an ultrasonic bath for 20 min. The cleaned glass substrate was treated with oxygen plasma (100 W, 30 min) to achieve a hydrophilic surface. A 50‐nm‐thick gold layer was thermally evaporated on the glass substrate for the inert electrode, using a shadow mask under 10^−6^ Torr. To prepare the solution precursor, 20 mg of dried Zr_6_O_4_OH_4_(OMc)_12_ powder was dissolved in 0.5 mL of 2‐methoxyethanol (anhydrous, Sigma Aldrich, USA). The Zr_6_O_4_OH_4_(OMc)_12_ thin film was fabricated by spin‐coating the solution onto the patterned substrate at 3000 rpm for 20 s. The films were then annealed at 100, 125, 150, 175, and 200 °C for 10 min to remove any residual solvent and to cross–link the cluster network. Using a shadow mask, a 50‐nm‐thick silver layer was thermally deposited and patterned as the active electrode of the memristors. The switching active area of the device was determined by the overlap of the top and bottom electrodes, which was 50 µm × 50 µm. For the lateral devices, a 50 nm‐gold electrode was first deposited onto a cleaned glass substrate and patterned via photolithography with a photoresist (PR) and a wet etching process using gold etchant (TFA, Transene). Without removing the PR, the 50 nm‐silver electrode was deposited on top and then lifted off to fabricate submicroscale lateral electrodes. The Zr_6_O_4_OH_4_(OMc)_12_ thin film was then spin‐coated onto the patterned substrate at 3000 rpm for 20 s, followed by annealing at 100, 150, and 200 °C for 10 min. For measuring the elastic modulus, 50 nm‐thick‐Zr_6_‐oxo thin films were prepared by spin‐coating under the same conditions and annealed at 100, 150, and 200 °C for 10 min.

### Characterization

The FT‐IR spectra of Zr_6_O_4_OH_4_(OMc)_12_ were obtained using an IRTracer‐100 (Shimadzu, Japan). The elastic modulus measurements were performed using force–distance (*F*–*D*) curve measurements on a commercial AFM system (Park Systems, NX‐10) with an aluminum‐coated reflective AFM tip (ContAl‐G, BudgetSensors, nominal spring constant 0.2 N m^−1^). The elastic modulus was extracted from the measured F‐D curve using the XEP (Park Systems) program with the Hertzian model and a conical tip shape. GIXRD was performed using an X'pert Pro (PANalytical, Netherlands) with Cu Kα radiation. The morphology of the lateral memristor device was analyzed using FE‐SEM (Hitachi S‐4700, Japan) at the MEMS Sensor Platform Center of SungKyunKwan University (SKKU). TEM images of the Ag/Zr_6_‐oxo/Au device were obtained using a TEM instrument (JEsM‐F200, Japan). The electrical properties of the memristor device were characterized by a Keithley 4200‐SCS instrument (Tektronix, USA) and an MST‐4000A probe station (MS TECH, Korea) under ambient conditions. For pulse measurement, a pulse generator (Tektronix, AFG3102C, USA) and an oscilloscope (Agilent Technologies, DSO X 3014A, USA) were used.

## Conflict of Interest

The authors declare no conflict of interest

## Supporting information



Supporting Information

## Data Availability

The data that support the findings of this study are available from the corresponding author upon reasonable request.

## References

[1] S. A. Chekol , S. Menzel , R. W. Ahmad , R. Waser , S. Hoffmann‐Eifert , Adv. Func. Mater. 2022, 32, 2111242.

[2] T. Wang , J. Meng , X. Zhou , Y. Liu , Z. He , Q. Han , Q. Li , J. Yu , Z. Li , Y. Liu , H. Zhu , Q. Sun , D. W. Zhang , P. Chen , H. Peng , L. Chen , Nat. Commun. 2022, 13, 7432. 36460675 10.1038/s41467-022-35160-1 PMC9718838

[3] W. Zhang , B. Gao , J. Tang , P. Yao , S. Yu , M. F. Chang , H. J. Yoo , H. Qian , H. Wu , Nat. Electron. 2020, 3, 371.

[4] J. Woo , J. H. Kim , J. P. Im , S. E. Moon , Adv. Intell. Syst. 2020, 2, 2000111.

[5] Y. Choi , S. Oh , C. Qian , J. H. Park , J. H. Cho , Nat. Commun. 2020, 11, 4595. 32929064 10.1038/s41467-020-17850-w PMC7490352

[6] X. Zhang , C. Wu , Y. Lv , Y. Zhang , W. Liu , Nano Lett. 2022, 22, 7246. 35984717 10.1021/acs.nanolett.2c02765

[7] Z. Peng , Z. Cheng , S. Ke , Y. Xiao , Z. Ye , Z. Wang , T. Shi , C. Ye , X. Wen , P. K. Chu , X. F. Yu , J. Wang , Adv. Func. Mater. 2023, 33, 2211269.

[8] M. Zaheer , A. U. R. Bacha , I. Nabi , J. Lan , W. Wang , M. Shen , K. Chen , G. Zhang , F. Zhou , L. Lin , M. Irshad , F. Faridullah , A. Arifeen , Y. Li , ACS Omega 2022, 7, 40911. 36406554 10.1021/acsomega.2c03893 PMC9670282

[9] A. S. Sokolov , H. Abbas , Y. Abbas , C. Choi , J. Semicond. 2021, 42, 013101.

[10] A. Sebastian , M. L. Gallo , R. Khaddam‐Aljameh , E. Eleftheriou , Nat. Nanotechnol. 2020, 15, 529. 32231270 10.1038/s41565-020-0655-z

[11] S. Y. Chun , Y. G. Song , J. E. Kim , J. U. Kwon , K. Soh , J. Y. Kwon , C. Y. Kang , J. H. Yoon , Adv. Mater. 2023, 35, 2302219. 10.1002/adma.202302219 37116944

[12] T. Y. Wang , J. L. Meng , M. Y. Rao , Z. Y. He , L. Chen , H. Zhu , Q. Q. Sun , S. J. Ding , W. Z. Bao , P. Zhou , D. W. Zhang , Nano Lett. 2020, 20, 4111. 32186388 10.1021/acs.nanolett.9b05271

[13] D. Kim , J. H. Park , D. S. Jeon , T. D. Dongale , T. G. Kim , J. Alloys Compd. 2021, 854, 157261.

[14] S. Chandrasekaran , F. M. Simanjuntak , R. Saminathan , D. Panda , T. Y. Tseng , Nanotechnolgy 2019, 30, 445205. 10.1088/1361-6528/ab3480 31341103

[15] J. H. Cha , B. C. Jang , J. Oh , C. Lee , S. Y. Yang , H. Park , S. G. Im , S. Y. Choi , Adv. Intell. Syst. 2022, 4, 2200018.

[16] H. J. Kim , T. H. Park , K. J. Yoon , W. M. Seong , J. W. Jeon , Y. J. Kwon , Y. Kim , D. E. Kwon , G. S. Kim , T. J. Ha , S. G. Kim , J. H. Yoon , C. S. Hwang , Adv. Func. Mater. 2019, 29, 1806278.

[17] H. L. Park , M. H. Kim , M. H. Kim , S. H. Lee , Nanoscale 2020, 12, 22502. 33174583 10.1039/d0nr06964g

[18] M. H. Kim , H. L. Park , M. H. Kim , J. Jang , J. H. Bae , I. M. Kang , S. H. Lee , npj Flex. Electron. 2021, 5, 34.

[19] K. Zhang , P. Ganesh , Y. Cao , ACS Appl. Mater. Interfaces 2023, 15, 21219. 37083295 10.1021/acsami.3c00371

[20] H. Kim , J. Lee , H. W. Kim , J. Woo , M. H. Kim , S. H. Lee , ACS Appl. Mater. Interfaces 2023, 15, 51444. 10.1021/acsami.3c13514 37874750

[21] J. Ryu , K. Park , D. P. Sahu , T. S. Yoon , ACS Appl. Mater. Interfaces 2024, 16, 26450. 38739419 10.1021/acsami.4c04874

[22] B. Li , W. Wei , L. Luo , M. Gao , Z. G. Yu , S. Li , K. W. Ang , C. Zhu , Adv. Electron. Mater. 2022, 8, 2200089.

[23] A. Mazumder , T. Ahmed , E. Mayes , S. A. Tawfik , S. P. Russo , M. X. Low , A. Ranjan , S. Balendhran , S. Walia , Adv. Electron. Mater. 2022, 8, 2100999.

[24] T. Hussain , H. Abbas , C. Youn , H. Lee , T. Boynazarov , B. Ku , Y. R. Jeon , H. Han , J. H. Lee , C. Choi , T. Choi , Adv. Mater. Technol. 2022, 7, 2100744.

[25] M. Son , G. H. Kim , O. Song , C. Park , S. Kwon , J. Kang , K. Ahn , M. G. Kim , Adv. Sci. 2024, 11, 2308188. 10.1002/advs.202308188 PMC11005697 38303575

[26] K. Ahn , G. H. Kim , S. J. Kim , J. Kim , G. S. Ryu , P. Lee , B. Ryu , J. Y. Cho , Y. H. Kim , J. Kang , H. Kim , Y. Y. Noh , M. G. Kim , Chem. Mater. 2022, 34, 10517.

[27] Y. Zhang , F. De Azambuja , T. N. Parac‐Vogt , Catal. Sci. Technol. 2022, 12, 3190.

[28] D. Van den Eynden , R. Pokratath , J. P. Mathew , E. Goossens , K. De Buysser , J. De Roo , Chem. Sci. 2023, 14, 573. 36741516 10.1039/d2sc05037d PMC9847641

[29] G. H. Kim , J. Park , J. W. Jo , S. Kim , J. Hong , S. K. Park , K. Ahn , K. J. Baeg , M. G. Kim , J. Alloys Compd. 2023, 955, 170194.

[30] G. Kickelbick , U. Schubert , Chem. Ber. 1997, 130, 473.

[31] W. Sun , B. Gao , M. Chi , Q. Xia , J. J. Yang , H. Qian , H. Wu , Nat. Commun. 2019, 10, 3453. 31371705 10.1038/s41467-019-11411-6 PMC6672015

[32] J. Kang , T. Kim , S. Hu , J. Kim , J. Y. Kwak , J. Park , J. K. Park , I. Kim , S. Lee , S. Kim , Y. Jeong , Nat. Commun. 2022, 13, 4040. 35831304 10.1038/s41467-022-31804-4 PMC9279478

[33] Y. Yang , P. Gao , S. Gaba , T. Chang , X. Pan , W. Lu , Nat. Commun. 2012, 3, 732. 22415823 10.1038/ncomms1737

[34] Y. Yang , P. Gao , L. Li , X. Pan , S. Tappertzhofen , S. Choi , R. Waser , I. Valov , W. D. Lu , Nat. Commun. 2014, 5, 4232. 24953477 10.1038/ncomms5232

[35] Y. Li , J. Chu , W. Duan , G. Cai , X. Fan , X. Wang , G. Wang , Y. Pei , ACS Appl. Mater. Interfaces 2018, 10, 24598. 29995376 10.1021/acsami.8b05749

[36] N. Ilyas , J. Wang , C. Li , H. Fu , D. Li , X. Jiang , D. Gu , Y. Jiang , W. Li , J. Mater. Sci. Technol. 2022, 97, 254.

[37] X. Dong , H. Sun , X. Lai , F. Yang , T. Ma , X. Zhang , J. Chen , Y. Zhao , J. Chen , X. Zhang , Y. Li , J. Phys. Chem. Lett. 2024, 15, 3668. 38535723 10.1021/acs.jpclett.4c00600

[38] B. Li , W. Wei , L. Luo , M. Gao , C. Zhu , Microelectron. J. 2024, 146, 106141.

[39] U. Jung , M. Kim , J. Jang , J. H. Bae , I. M. Kang , S. H. Lee , Adv. Sci. 2023, 11, 2307494. 10.1002/advs.202307494 PMC10916635 38087893

[40] N. Raeis‐Hosseini , J. Noh , J. Shin , J. Rho , ACS Appl. Electron. Mater. 2024, 6, 3501.

[41] S. Oh , H. Kim , S. E. Kim , M. H. Kim , H. L. Park , S. H. Lee , Adv. Intell. Syst. 2023, 5, 2200272.

[42] D. P. Sahu , P. Jetty , S. N. Jammalamadaka , Nanotechnology 2021, 32, 155701. 33412536 10.1088/1361-6528/abd978

[43] K. Kim , D. C. Kang , Y. Jeong , J. Kim , S. Lee , J. Y. Kwak , J. Park , G. W. Hwang , K. S. Lee , B. K. Ju , J. Alloys Compd. 2021, 884, 161086.

[44] Y. J. Jeon , H. An , Y. Kim , Y. P. Jeon , T. W. Kim , Appl. Surf. Sci. 2021, 567, 150748.

[45] T. Li , H. Yu , Z. Xiong , Z. Gao , Y. Zhou , S. T. Han , Mater. Horiz. 2021, 8, 2041. 34846481 10.1039/d1mh00315a

[46] T. J. Lee , S. K. Kim , T. Y. Seong , Sci. Rep. 2020, 10, 5761. 32238846 10.1038/s41598-020-62642-3 PMC7113278

[47] Y. Li , L. Loh , S. Li , L. Chen , B. Li , M. Bosman , K. W. Ang , Nat. Electron. 2021, 4, 348.

[48] W. Ahn , H. B. Jeong , J. Oh , W. Hong , J. H. Cha , H. Y. Jeong , S. Y. Choi , Small 2023, 19, 2300223. 10.1002/smll.202300223 37093184

[49] M. J. Josline , S. Ghods , S. Kosame , J. H. Choi , W. Kim , S. Kim , S. Chang , S. H. Hyun , S. I. Kim , J. Y. Moon , H. G. Park , S. B. Cho , H. Ju , J. H. Lee , Small 2024, 20, 2307276. 10.1002/smll.202307276 38196162

