# Supplementary material for: Flexible Synaptic Memristors With Controlled Rigidity in Zirconium‐Oxo Clusters for High‐Precision Neuromorphic Computing
Source: Adv Sci (Weinh). 2025 Jan 24;12(11):2412289. doi: 10.1002/advs.202412289 (PMC11923897; doi:10.1002/advs.202412289)
Supplement: Supplementary file 1 — Supporting Information [file ADVS-12-2412289-s001.docx]

Supporting Information

Flexible Synaptic Memristors with Controlled Rigidity in Zirconium-Oxo Clusters for High-Precision Neuromorphic Computing

Jae-Hyeok Cho, Suk Yeop Chun, Ga Hye Kim, Panithan Sriboriboon, Sanghee Han, Seung Beom Shin, Jeehoon Kim, San Nam, Yunseok Kim, Yong-Hoon Kim^*,^ Jung Ho Yoon^*^, Myung-Gil Kim^*^


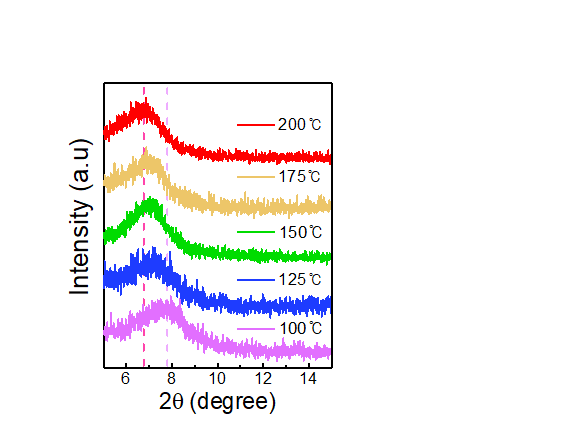


**Figure S1.** Grazing incidence X-ray diffraction (GIXRD) patterns of the Zr_6_-oxo cluster thin film under various thermal annealing conditions.


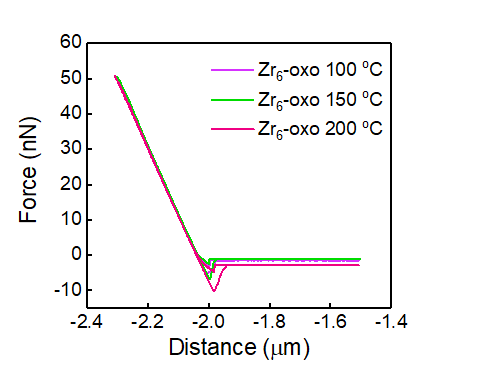


**Figure S2**. AFM force-distance curves for Zr_6_-oxo thin films annealed at different temperatures.


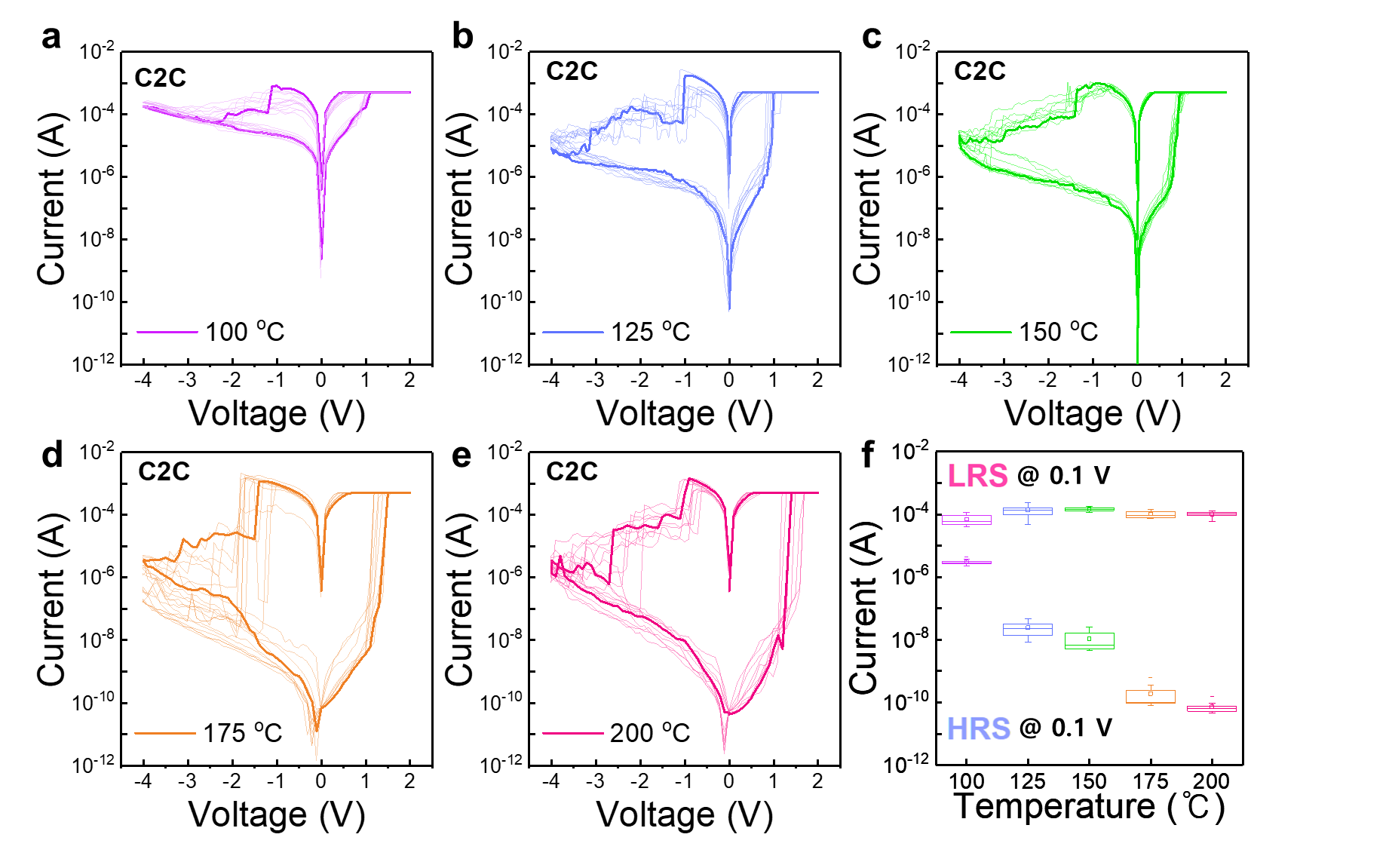


**Figure S3.** *I-V* characteristics of the memristor device under five different annealing conditions: (a) 100 °C, (b) 125 °C, (c) 150 °C, (d) 175 °C, and (e) 200 °C. (f) Box plots of the low-resistance state (LRS) and high-resistance state (HRS) current levels at 0.1 V for each annealing condition.

**
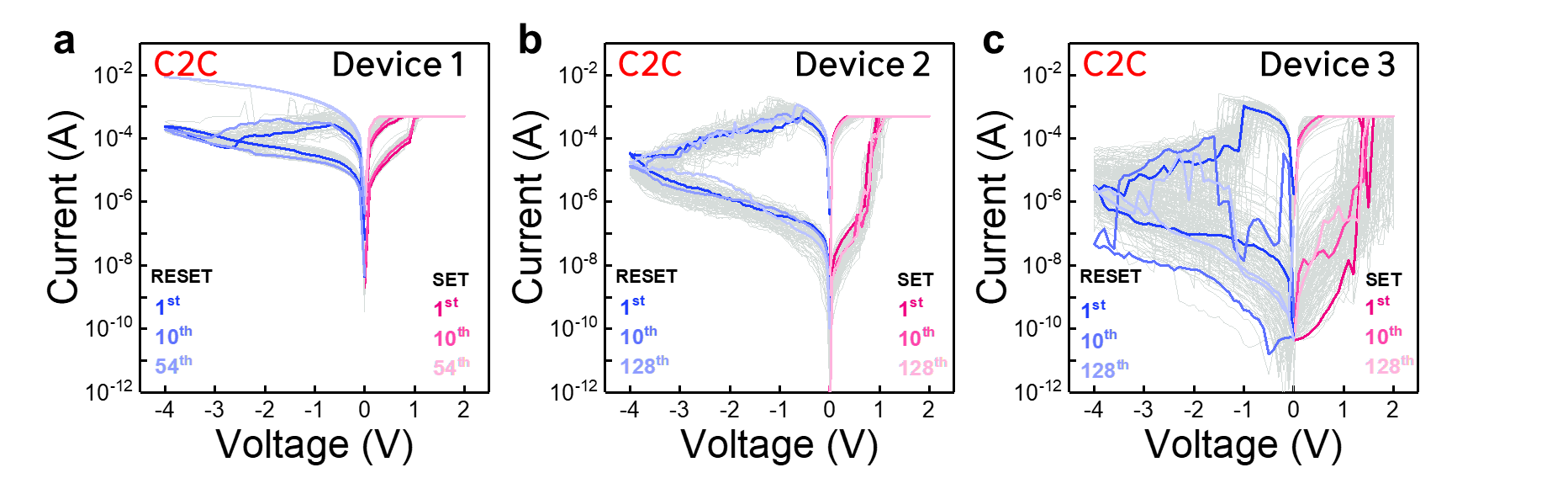
**

**Figure S4**. *I-V* curves of Ag/Zr_6_-oxo/Au device under various annealing conditions for consecutive DC sweep cycles. a) device 1: Annealed at 100 °C, b) device 2 : Annealed at 150 °C, c) device 3 : Annealed at 200 °C.

**
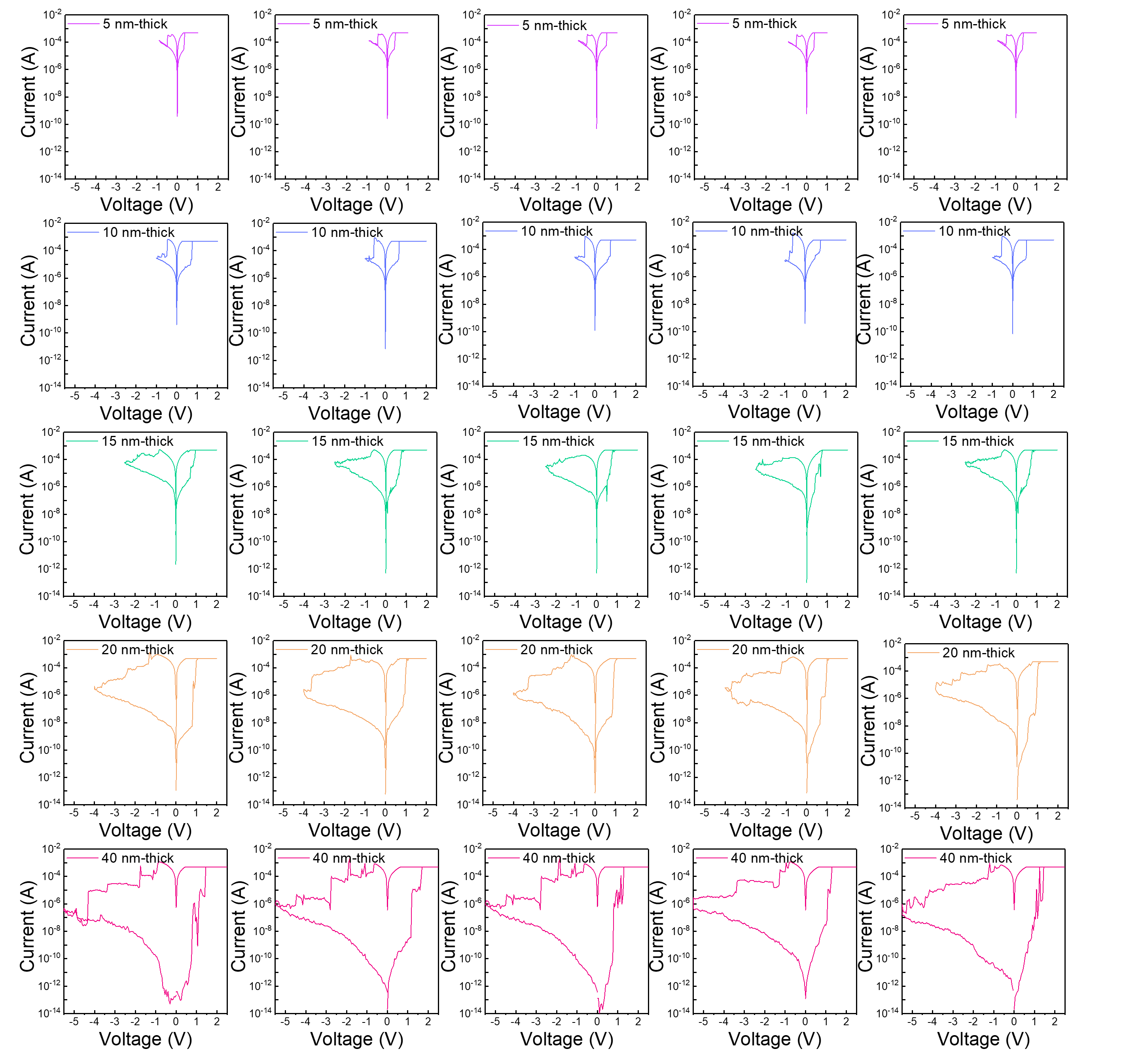
**

**Figure S5.** *I-V* characteristics of 5 different memristor devices fabricated with zirconium-oxo cluster layers of varying thicknesses: 5 nm, 10 nm, 15 nm, 20 nm, and 40 nm, annealed at 150 °C.


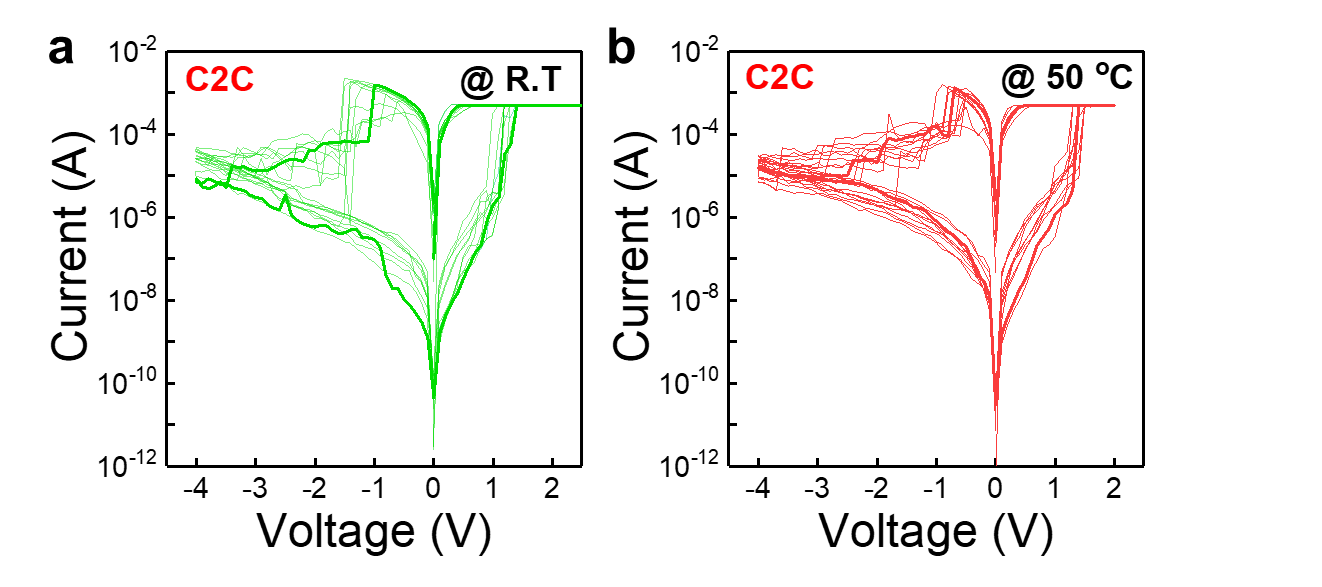


**Figure S6**. *I-V* characteristics of device 2 measured under two temperature conditions: room temperature (R.T.) and 50 °C.


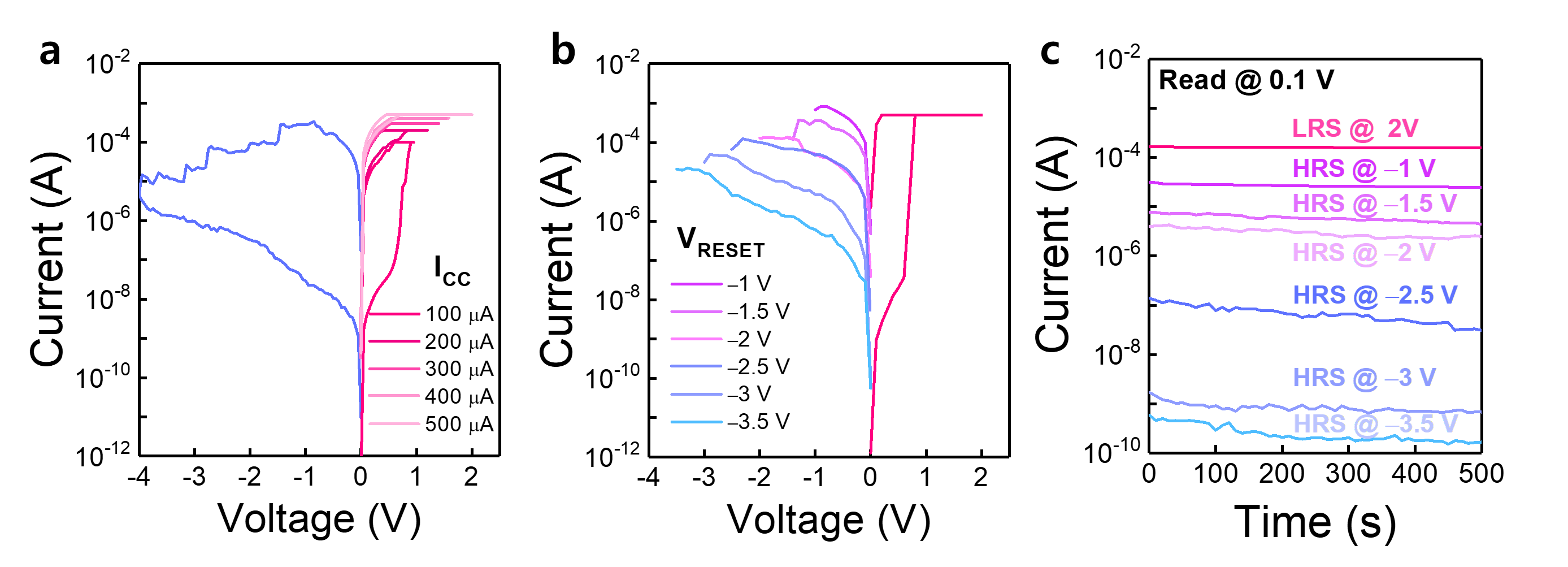


**Figure S7.** a) *I-V* curves of device 2 showing multiple LRS by controlling the compliance current (I_CC_). b) *I-V* curves of device 2 showing partial stop reset processes at various reset voltages from 0 to −1 V, −1.5 V, −2 V, −2.5 V, −3, and −3.5 V. c) Retention characteristics of each resistance state.


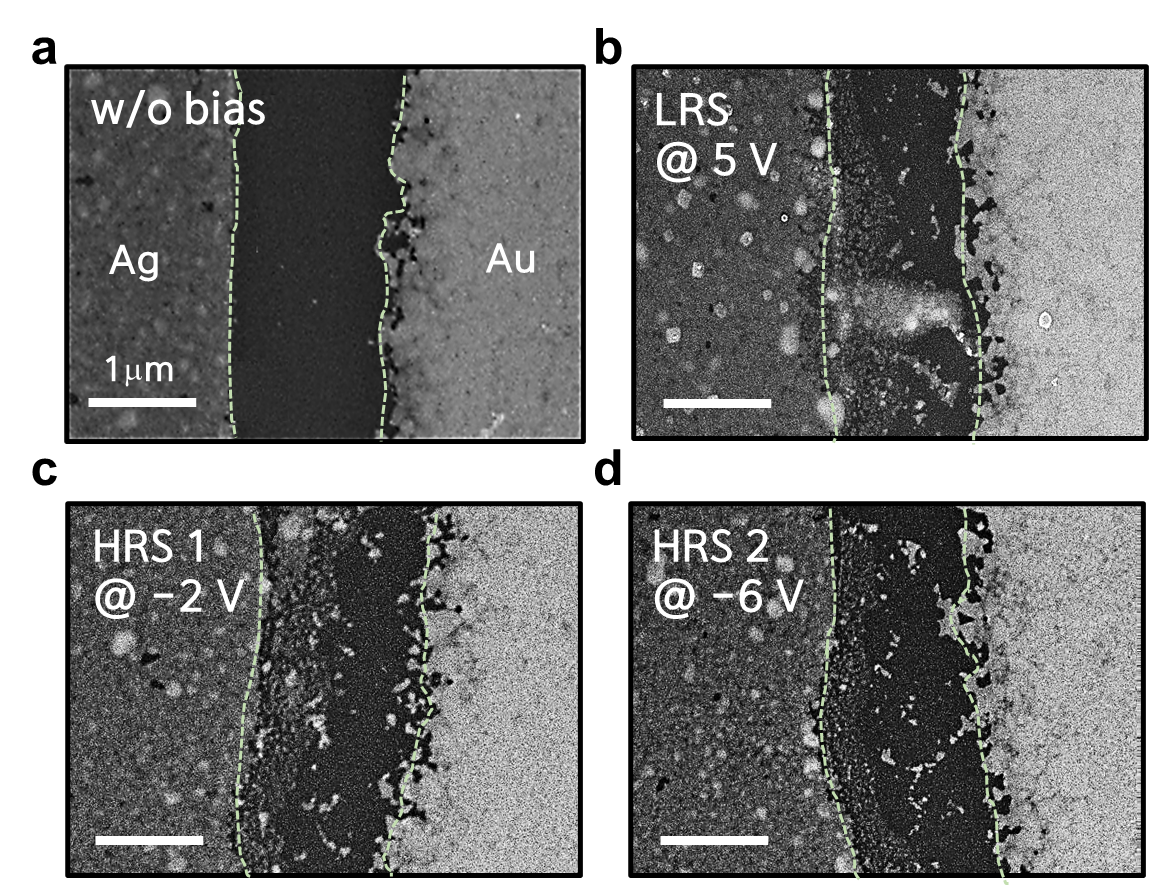


**Figure S8**. FE-SEM images of a lateral device under various voltage bias conditions: (a) without applied bias (w/o bias), (b) at 5 V for the SET process, at (c) −2 V, and (d) −6 V for the RESET process.


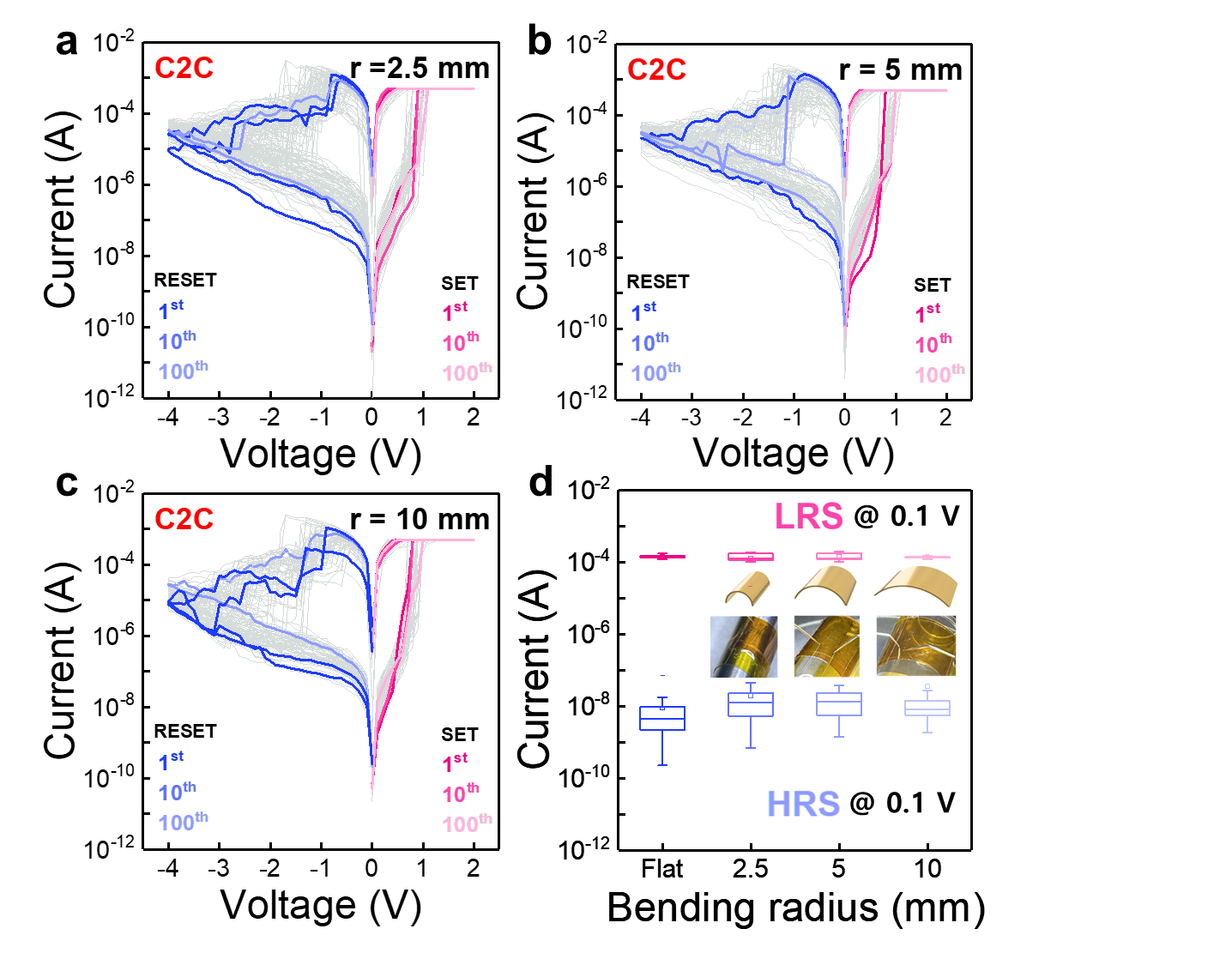


**Figure S9**. *I-V* characteristics of the device measured over 100 cycles at bending radii of (a) 2.5 mm, (b) 5 mm, and (c) 10 mm. (d) Box plots of the current levels at 0.1 V in the low-resistance state (LRS) and high-resistance state (HRS) for flat and bent conditions with radii of 2.5, 5, and 10 mm. The insets show photographs of the device under the corresponding bending conditions.


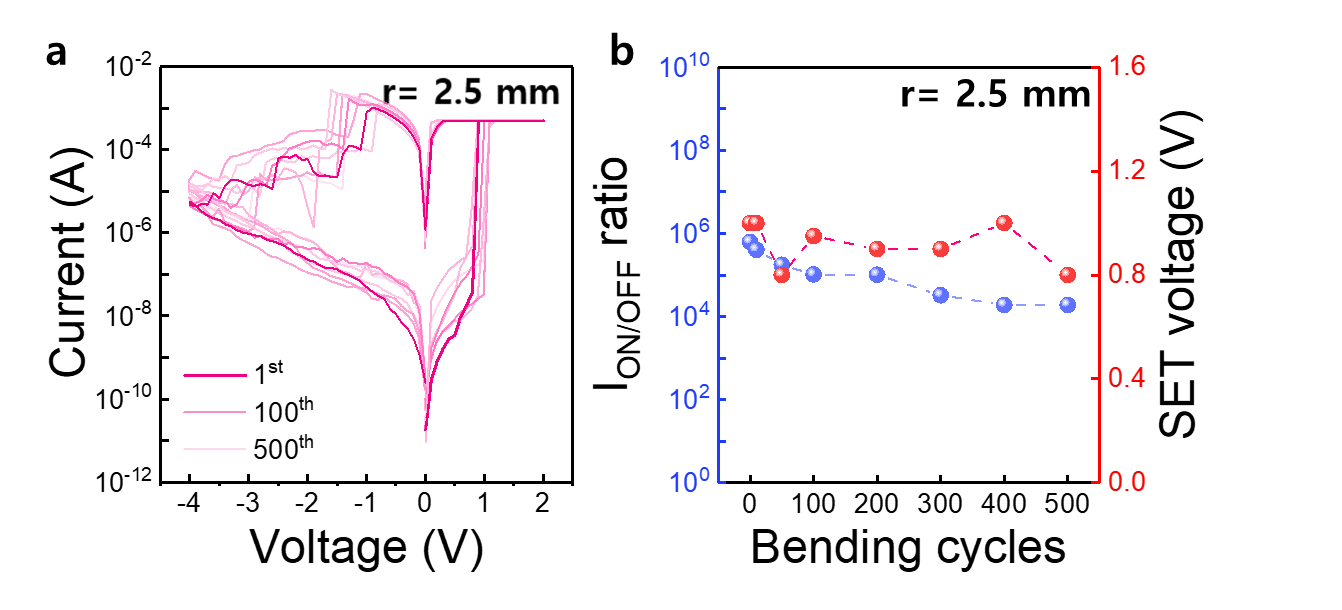


**Figure S10**. a) *I-V* characteristics of the flexible memristor device under a bending radius of 2.5 mm, over 500 consecutive bending cycles.b) ON/OFF ratio and SET voltage of flexible device after 500 bending cycles with bending radius of 2.5 mm.

**
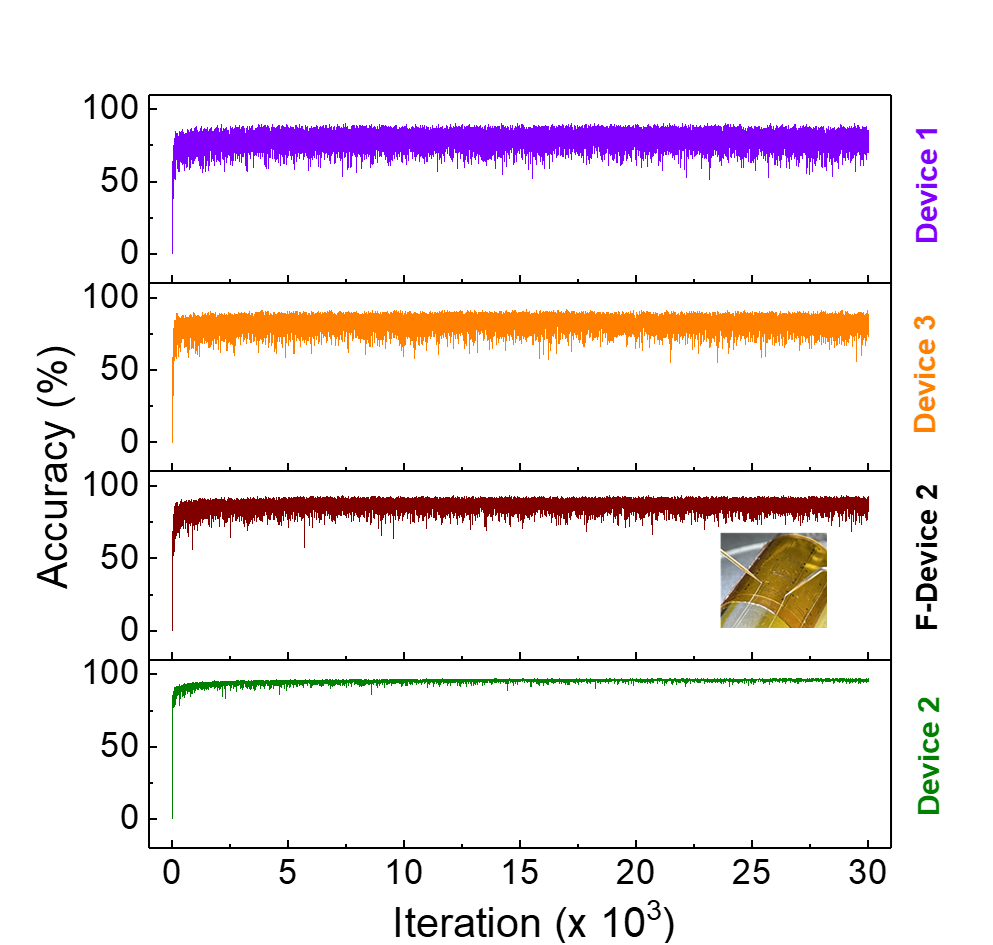
**

**Figure S11.** Comparison of pattern recognition accuracy under different conditions after 30,000 iterations.

**Table S1.** Summary of device parameters for flexible memristors.

| Structure | Mechanism | Operating voltage  (V) | Bending radius  (mm) | Bending cycles | linearity factors (ν_P_/ν_D_) | Pulse type (number of pulse) | Ref. |
| --- | --- | --- | --- | --- | --- | --- | --- |
| Ag/Al_2_O_3_ NP:PI/ITO | ECM | 3/−5 | 10 | 400 | N/A | N/A | ^[1]^ |
| Mg/collagen/ITO | ECM | 3/−3 | 7 | N/A | N/A | N/A | ^[2]^ |
| Al/IAO/In2O3/US | VCM | 3/−2 | 4.2 | 500 | 3.66/2.52 | Identical (50) | ^[3]^ |
| ITO/BN/TaN | VCM | 2/−2.5 | 10 | N/A | 10.37/9.89 | Identical (50) | ^[4]^ |
| Au/SiO*_x_*/Al(9%):TiO*_x_*/Ti | VCM | 1/−2 | 5 | N/A | 1.85/1.47 | Identical (70) | ^[5]^ |
| Ag/albumen:CuO  /ITO | ECM | 6/−6 | 16 | 500 | 2.53/2.85 | nonidentical (50) | ^[6]^ |
| Ag/Mn-ZnO/ITO | ECM | 4/−4 | 10 | N/A | N/A | N/A | ^[7]^ |
| Al/PEDOT:PSS/ITO | VCM | 1/−1 | 2.5 | 1000 | 1.51/2.20 | Identical (50) | ^[8]^ |
| Ag/PEI-AgClO_4_/Pt | ECM | 2/−2 | 5.0 | 200 | N/A | N/A | ^[9]^ |
| Ag/Zr_6_-oxo/Au | ECM | 2/−4 | 2.5 | 500 | 1.11/1.29 | Identical (50) | This work |

**Table S2.** Summary of device parameters of ECM-based memristor.

| Structure | Mechanism | I_on/off_ | V_SET_/V_RESET_ | *Retention* | Endurance | linearity factors (ν_P_/ν_D_) | Pulse type (number of pulse) | Ref. |
| --- | --- | --- | --- | --- | --- | --- | --- | --- |
| Ag/Ti:a-Si/Au | ECM | <10^2^ | 0.3/−0.4 | 600 | 6 × 10^3^ | 2.28/2.46 | Identical (200) | ^[10]^ |
| Ag/NiO/Pt | ECM | 10^2^ | 0.39/−0.4 | 10^4^ | 10^2^ | 2.27/2.91 | Identical (50) | ^[11]^ |
| Ag/STO:Ag/SiO_2_/*p*++ Si | ECM | <10^3^ | 2.4/−2.6 | 10^4^ | 5 × 10^3^ | 1.60/1.17 | Identical (30) | ^[12]^ |
| Mo/MoO_x_/Ag | ECM | 10^4^ | −1.56/1.37 | 10^4^ | 10^2^ | 5.57/ N/A | Identical (50) | ^[13]^ |
| Ti/CuI/Au | ECM | 10^5^ | 0.67/−0.34 | 6 × 10^4^ | N/A | 2.22/2.42 | Identical (50) | ^[14]^ |
| Ag/PEDOT:PSS/Au | ECM | 10^2^ | 0.58/−0.42 | 5 × 10^3^ | 500 | 1.02/1.48 | Identical (30) | ^[15]^ |
| Mg/collagen/ITO | ECM | 20 | 1.44/−1.0 | 10^4^ | 120 | 2.40/9.31 | Identical (32) | ^[16]^ |
| Ag/PVA/ITO | ECM | <10^2^ | 1.4/−1.8 | 10^4^ | 300 | 1.09/1.31 | nonidentical  (16) | ^[17]^ |
| Ag/GO/ITO | ECM | 22 | 1.0/−2.5 | 10^4^ | 450 | 1.83/4.04 | Identical (45) | ^[18]^ |
| Ag/SPE-SiGe/*p*-Si | ECM | <10^2^ | 2.9/−1.8 | 2 × 10^3^ | N/A | 6.16/3.40 | Identical (30) | ^[19]^ |
| Ag/UiO-66@PVA/FTO | ECM | 10 | 1.5/−1.2 | 1.3 × 10^3^ | 500 | 10.51/22.36 | Identical (100) | ^[20]^ |
| Ag/COF-5/ITO | ECM | 10^2^ | 1.0/−1.0 | 10^5^ | 10^3^ | 1.0/4.9 | Identical (50) | ^[21]^ |
| Ag/SrVO_x_/Ti/Pt | ECM | 10^3^ | 0.4/−0.3 | 2 × 10^4^ | 10^2^ | 1.13/4.4 | Identical (40) | ^[22]^ |
| Ti/PdSe_2_/Au | ECM | 10^3^ | 0.75/−0.65 | 9 × 10^4^ | N/A | 4.06/5.21 | Identical (100) | ^[23]^ |
| Cu/Ti/MoS_2_/Al_2_O_3_/Au | ECM | 10^3^ | 0.33/−0.22 | 10^4^ | 10^3^ | 1.17/1.13 | nonidentical (50) | ^[24]^ |
| Ag/Zr_6_-oxo/Au | ECM | 10^4^ | 0.95/−3.60 | 10^4^ | 10^4^ | 1.04/1.05 | Identical  (50) | This work |

**References**

[1] C. Wu, T. W. Kim, T. Guo, F. Li, D. U. Lee, J. J. Yang, *Adv. Mater.* **2017**, 29(10), 1602890.

[2] N. Raeis-Hosseini, Y. Park, J.-S. Lee, *Adv. Func. Mater.* **2018**, 28(31), 1800553.

[3] W. Zhang, Y. Mao, W. Duan, *Phys. Status Solidi - Rapid Res. Lett.* **2019**, 13(6), 1900016.

[4] *J.-L. Meng, T.-Y. Wang, Z.-Y. He, L. Chen, H. Zhu, L. Ji, Q.-Q. Sun, S.-J. Ding, W.-Z. Bao, P. Zhou, D. W. Zhang, Mater. Horiz.* **2021**, 8(2), 538-546.

[5] H. Song, S. Hu, Y. Liu, R. Bao, J. Liu, X. Zhong, J. Wang, *Phys. Status Solidi A* **2024**, 221(6), 2300827.

[6] T. Guo, J. Ge, B. Sun, K. Pan, Z. Pan, L. Wei, Y. Yan, Y. N Zhou, Y. A. Wu, *Adv. Elec. Mater.* **2022**, 8(10), 2200449.

[7] Q. Xue, T. Hang, J. Liang, C.-C Chen, Y. Wu, H. Ling, M. Li, *J. Mater. Sci. Technol.* **2022**, 119, 123-130.

[8] X. Luo, J. Ming, J. Gao, J. Zhuang, J. Fu, Z. Ren, H. Ling, L. Xie, *Front. Neurosci.* **2022**, 16, 1016026.

[9] X. Zhang, C. Wu, Y. Lv, Y. Zhang, W. Liu, *Nano Lett.* **2022**, 22, 7246−7253.

[10] J. Kang, T. Kim, S. Hu, J. Kim, J. Y. Kwak, J. Park, J. K. Park, I. Kim, S. Lee, S. Kim, Y. Jeong, *Nat. Commun.* **2022**, 13, 4040.

[11] Y. Li, J. Chu, W. Duan, G. Cai, X. Fan, X. Wang, G. Wang, Y. Pei, *ACS Appl. Mater. Interfaces* **2018**, 10, 24598.

[12] N. Ilyas, J. Wang, C. Li, H. Fu, D. Li, X. Jiang, D. Gu, Y. Jiang, W. Li, *J. Mater. Sci. Technol.* **2022**, 97, 254.

[13] X. Dong, H. Sun, X. Lai, F. Yang, T. Ma, X. Zhang, J. Chen, Y. Zhao, J. Chen, X. Zhang, Y. Li, *J. Phys. Chem. Lett.* **2024**, 15, 3668.

[14] B. Li, W. Wei, L. Luo, M. Gao, C. Zhu, *Microelectron. J.* **2024**, 106141.

[15] U. Jung, M. Kim, J. Jang, J. H. Bae, I. M. Kang, S. H. Lee, *Adv. Sci.* **2023**, 11, 2307494.

[16] N. Raeis-Hosseini, J. Noh, J. Shin, J. Rho, *ACS Appl. Electron. Mater.* **2024,** 6, 3501.

[17] S. Oh, H. Kim, S. E. Kim, M.-H. Kim, H.-L. Park, S.-H. Lee, *Adv. Intell. Syst.* **2023**, 5, 2200272.

[18] D. P. Sahu, P. Jetty, S. N. Jammalamadaka, *Nanotechnology* **2021**, 32, 155701.

[19] K. Kim, D. C. Kang, Y. Jeong, J. Kim, S. Lee, J. Y. Kwak, J. Park, G. W. Hwang, K.-S. Lee, B.-K. Ju, *J. Alloys Compd.* **2021**, 884, 161086.

[20] Y. J. Jeon, H. An, Y. Kim, Y. P. Jeon, T. W. Kim, *Appl. Surf. Sci.* **2021**, 567, 150748.

[21] T. Li, H. Yu, Z. Xiong, Z. Gao, Y. Zhou, S.-T. Han, *Mater. Horiz.* **2021**, 8, 2041.

[22] T.-J. Lee, S.-K. Kim, T.-Y. Seong, *Sci. Rep.* **2020**, 10, 5761.

[23] Y. Li, L. Loh, S. Li, L. Chen, B. Li, M. Bosman, K.-W. Ang, *Nat. Electron.***2021**, 4, 348.

[24] W. Ahn, H. B. Jeong, J. Oh, W. Hong, J. H. Cha, H. Y. Jeong, S. Y. Choi, *Small* **2023**, 19, 2300223.
